# Impact of a Specific Collagen Peptide Food Supplement on Periodontal Inflammation in Aftercare Patients—A Randomised Controlled Trial

**DOI:** 10.3390/nu14214473

**Published:** 2022-10-25

**Authors:** Yvonne Jockel-Schneider, Peggy Stoelzel, Jeanine Hess, Imme Haubitz, Stefan Fickl, Ulrich Schlagenhauf

**Affiliations:** Department of Periodontology, University Hospital Wuerzburg, Pleicherwall 2, D-97070 Wuerzburg, Germany

**Keywords:** collagen, peptide fragment, bleeding on probing, gingival, food supplement, periodontitis, gingivitis

## Abstract

**Background:** This controlled clinical trial evaluated the impact of a specific collagen peptide food supplement on parameters of periodontal inflammation in aftercare patients. **Methods:** A total of 39 study patients were enrolled. At baseline, bleeding on probing (BoP; primary outcome), gingival index (GI), plaque control record (PCR), recession (REC) and probing pocket depth (PPD) for the calculation of the periodontal inflamed surface area (PISA) were documented. After subsequent professional mechanical plaque removal (PMPR), participants were randomly provided with a supply of sachets containing either a specific collagen peptide preparation (test group; *n* = 20) or a placebo (placebo group; *n* = 19) to be consumed dissolved in liquid once daily until reevaluation at day 90. **Results:** PMPR supplemented with the consumption of the specific collagen peptides resulted in a significantly lower mean percentage of persisting BoP-positive sites than PMPR plus placebo (test: 10.4% baseline vs. 3.0% reevaluation; placebo: 14.2% baseline vs. 9.4% reevaluation; effect size: 0.86). Mean PISA and GI values were also reduced compared to baseline, with a significant difference in favor of the test group (PISA test: 170.6 mm^2^ baseline vs. 53.7 mm^2^ reevaluation; PISA placebo: 229.4 mm^2^ baseline vs. 184.3 mm^2^ reevaluation; GI test: 0.5 baseline vs. 0.1 reevaluation; GI placebo: 0.4 baseline vs. 0.3 reevaluation). PCR was also significantly decreased in both experimental groups at revaluation, but the difference between the groups did not reach the level of significance. **Conclusions:** The supplementary intake of specific collagen peptides may further enhance the anti-inflammatory effect of PMPR in periodontal recall patients.

## 1. Introduction

Professional mechanical plaque control complemented by efficacious oral hygiene is the established standard of supportive periodontal aftercare (SPC) [1]. Its validity has been verified by numerous studies [2,3,4,5,6]. Nevertheless, even in the majority of successfully treated cases, a small but clinically relevant amount of periodontal inflammation persists, reflected by the number of periodontal pockets with remaining bleeding on probing (BoP), which is associated with an increased risk of disease recurrence or progression [7,8,9,10]. As the occurrence of disease-promoting bacterial dysbiosis is strongly promoted by an increased inflammatory status of the host, the use of adjuvant therapeutics that modulate inflammation at the local and/or systemic level is advocated to further improve the outcome of mechanical plaque control [11,12]. This may involve the systemic or local administration of drugs that either inhibit the inflammatory response or actively promote its resolution [13], as well as targeted changes in the daily diet of patients [14,15,16,17,18] or the consumption of food supplements such as probiotics or omega-3 fatty acids [19,20,21,22]).

The list of known anti-inflammatory food supplements also includes certain collagen peptides that have been successfully evaluated as therapeutic adjuncts in the control of rheumatoid arthritis, which shares common risk factors and aetiological pathways with periodontitis [23,24,25].

In animal and human interventional trials, the consumption of collagen peptides high in hydroxyproline, glycine and proline resulted in improved wound healing and the remediation of impaired immune system function [26,27]. The preventive addition of glycine to the culture media of isolated intestinal epithelial cells mitigated the negative impact of inflicted oxidative stress at the cellular level [28]. In addition, high glycine concentrations in the cell culture media of porcine intestinal epithelial cells improved the barrier function of tight junctions [29].

Furthermore, the addition of specific collagen hydrolysates to the culture media of bacteria derived from the oral or intestinal microbiota had a significant impact on the selective microbial utilization of amino acids [30,31]. It also favoured the synthesis of short-chain fatty acids, which, among other benefits, are known to enhance the maturation of regulatory T-cells and the sealing of epithelial barriers [32].

Based on these in vitro observations, the oral administration of specific collagen peptides in patients suffering from chronic osteoarthritis has already entered clinical practice and resulted in a relevant reduction in pain and functional impairments [33,34,35].

It was the aim of this study to assess the impact of the adjuvant consumption of a specific, commercially available collagen peptide food supplement on parameters of periodontal inflammation in a cohort of aftercare patients.

## 2. Material and Methods

### 2.1. Study Design

This investigation was designed as a two-arm, double blind, parallel group, randomised placebo-controlled trial with a 3-month observation period.

The study protocol was established in accordance with the Helsinki Declaration of 1975, as revised in 2013, and the criteria of good clinical practice. It was approved by the ethics committee of the University of Wuerzburg (file # 37/18-me) and registered with clinicaltrials.gov (identifier # NCT03765125). All participants were informed about the objectives and risks in personal interviews and were included in the study after having given written informed consent.

### 2.2. Study Population

Study patients were recruited from treated chronic periodontitis patients who were in regular supportive aftercare at the Department of Periodontology of the University Hospital of Wuerzburg. The study was conducted between 21 September 2018 (first patient in) and 26 August 2019 (last patient out).

### 2.3. Eligibility Criteria

Eligibility criteria for study participation were a minimum of 10 natural teeth, a history of treated chronic periodontitis followed by regular supportive periodontal aftercare and the presence of mild-to-moderate chronic gingivitis equal to a gingival index (GI) score of 1 or 2 at a minimum of three teeth.

### 2.4. Exclusion Criteria

Exclusion criteria were the manifestation of inflammatory oral mucosal diseases other than periodontitis, the presence of xerostomia, pregnancy, acute infections, smoking > 10 cigarettes per day, the intake of antibiotics and/or anti-inflammatory drugs < 4 weeks prior to screening and the presence of chronic systemic diseases (e.g., diabetes).

### 2.5. Experimental Preparations

Both experimental preparations to be consumed by the study patients were manufactured and supplied by GELITA AG (Eberbach, Germany).

The test preparation (Verisol^®^B, GELITA AG, Eberbach, Germany) consisted of a mixture of specific, bioactive collagen peptides with an average molecular weight of approximately 2 kDa and an average particle size of about 150 μm. The placebo preparation consisted of silica (Evonik, Germany) being identical regarding taste, smell and color, as well as particle size and consistence to the test preparation. Both experimental preparations were manufactured in compliance with the European Community (EC) regulation 852/2004 on the hygiene of foodstuffs and packaged in individual code-labelled sachets containing the daily dosage of 5 g. According to the manufacturer’s information, the daily dose of the collagen hydrolysate administered for improving skin conditions is usually 2.5 g [36,37]. Evidence from other medical fields concerning the therapy of knee joint pain [38] or osteoporosis [39] as well as unpublished pretrial data on the impact of collagen peptides on gingivitis suggested that doubling the dosage to 5 g daily may be advisable.

### 2.6. Sequence of Study Intervention

**Figure 1:**
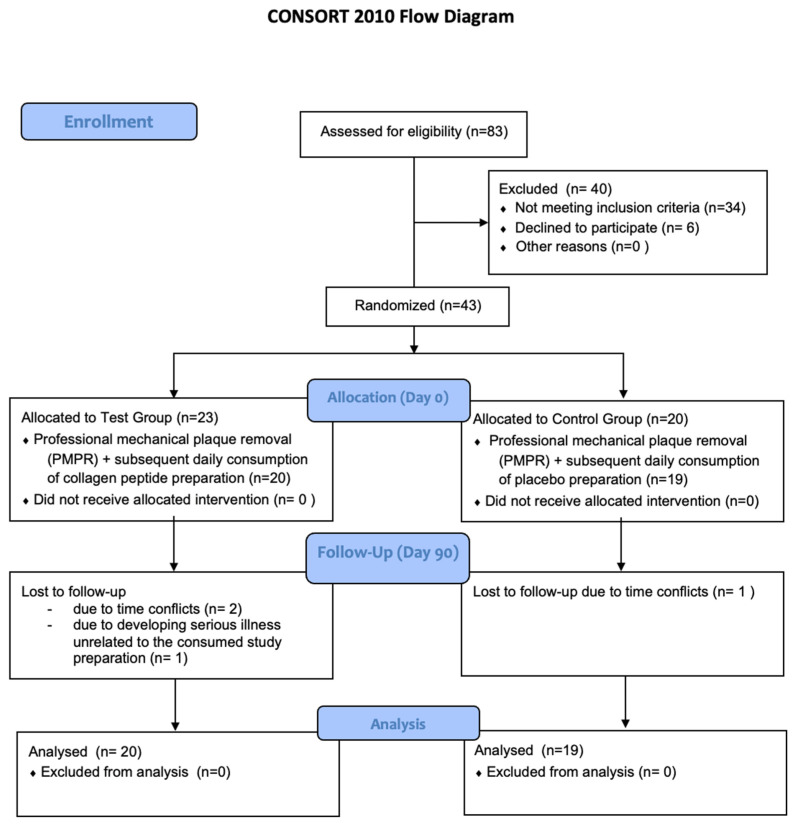
Recruitment, drop-outs and protocol violations during the study observation period.

The sequence of the study intervention is schematically depicted in Figure 1.

#### 2.6.1. Visit 1 Baseline (Day 0)

At baseline, the parameters bleeding on probing (BoP; primary outcome), probing pocket depth (PPD), gingival recession (REC), gingival index (GI) and plaque control record (PCR) were assessed on all teeth. A general health profile of the participants, including current intake of medications and frequency of smoking, was documented using a questionnaire. Assessment of BoP and calculation of the Periodontal Inflamed Surface Area (PISA)-Index, REC, PPD and BoP were recorded at six sites per tooth (mesio-buccal, buccal, disto-buccal, disto-oral, oral, mesio-oral) using a manual periodontal probe (UNC-15) (Hu-Friedy, Chicago, IL, USA), measured to the nearest millimeter. Any bleeding spot appearing within 30 s after probing was recorded as a BoP-positive site.

Subsequently, all teeth were cleaned supra- and subgingivally by standard PMPR using hand instruments and ultrasonic scalers followed by air polishing with a low-abrasive erythritol cleaning powder.

Finally, all participants randomly received a supply of code-labelled sachets containing either the experimental collagen peptide preparation (test group; *n* = 20) or the placebo preparation (control group; *n* = 19).

The preparations had to be consumed once daily suspended in cold or warm beverages or other aqueous foods. Consumption time could be chosen freely at one’s own discretion, regardless of the timing of oral hygiene or meals.

Unused sachets should be returned at the follow-up appointment.

#### 2.6.2. Visit 2 Reevaluation (Day 90)

At the follow-up appointment on day 90, BoP, PPD, REC, GI and PCR were recorded again as described before, and unused sachets returned by the participants were collected.

The total amount of periodontal inflammation present in each patient was calculated from the recorded BoP, REC and PPD scores using the PISA-Index [40].

#### 2.6.3. Assessment of Gingival Index (GI)

The GI is used to assess the degree of inflammation of the gingiva. In the variation of the GI according to Lobene [41] applied here, the degree of inflammation was assessed visually on the basis of colour and surface changes as well as swelling. The GI was recorded on the buccal aspect of all teeth.

#### 2.6.4. Assessment of PCR

The extent of plaque coverage of the teeth was quantified using the Plaque Control Record (PCR) [42]. Briefly, the crown of a tooth is divided into four quadrants, and the presence/absence of bacterial plaque on each quadrant is assessed. The percentage of tooth quadrants covered by bacterial plaque is then calculated. Before recording the PCR all tooth surfaces were dried by an air-syringe to make adhering bacterial plaque visible. 

#### 2.6.5. Assessment of Consumption Compliance

Consumption compliance was calculated by subtracting the number of unused, returned sachets from the total number of sachets provided at baseline. Consumption of the study preparation was considered to be per protocol when ≤10% of the supplied sachets were returned unused.

#### 2.6.6. Adverse Events

The study participants were instructed to report any adverse events immediately, whether related or unrelated to the consumption of the experimental preparations, and were specifically asked at the end of the study about the occurrence of adverse events during the trial.

#### 2.6.7. Blinding, Randomisation and Examiner Calibration

All clinical examinations were conducted by one experienced clinician who was blinded to the group assignment of the study patients.

To minimize intra-examiner variability of periodontal measurements, examiner calibration was performed according to Hefti et al. and Grossi et al. [43,44]. Random allocation of the experimental preparation was ensured using a computer-generated randomisation list with a block size of four.

### 2.7. Statistical Analysis

#### 2.7.1. Primary Study Outcome and Null Hypothesis

The primary outcome of this trial was defined as the change in the percentage of BoP-positive probing sites between baseline and the end of the study with the null hypothesis of no significant differences between both experimental groups.

#### 2.7.2. Secondary Study Outcomes

Secondary study outcomes were differences in the recorded mean scores of GI, PCR and PISA (calculated from PPD and REC) at baseline and day 90.

#### 2.7.3. Sample Size Calculation

In order to be able to verify a difference of 20% in the reduction of BoP-positive sites between the test and control group at day 90 with a power of 90%, significance level α = 0.05 and an estimated standard deviation of ±20%, sample size calculation resulted in a group size of 2 × 19 study patients when using the Mann–Whitney *U* test (G-Power, Version 3.1.9.4).

#### 2.7.4. Statistical Data Analysis

Data distribution was examined using probit diagrams with Lillefors limits. As most of the data deviated from a normal distribution, rank tests were used throughout. The difference between study groups was analysed with the Mann–Whitney *U* test. The change from baseline to post-intervention within groups was analysed with a Wilcoxon signed-rank test. Dichotomous values were tested with Fisher’s exact test.

All data are presented as mean ± SD or median with (25% tail, 75% tail); WinMEDAS statistical software package was used for all statistical analyses. All the tests in the descriptive analysis were conducted as two-sided tests. The significance level was set to *p* ≤ 0.05. Assessment of the study data was performed as an intention-to-treat analysis.

## 3. Results

### 3.1. Recruitment, Drop-Outs, Protocol Violations

From a total of 83 screened patients, 49 were recruited for study participation. Six of them withdrew their consent prior to baseline examination. Finally, 43 patients were randomly assigned to the test (*n* = 23) or the control group (*n* = 20). After the baseline visit, three study patients (two test /one control) left the study due to time conflicts, and one study participant (test group) left due to developing serious illness unrelated to the consumed study preparation.

The remaining 39 patients (test *n* = 20 and control *n* = 19) completed the study per protocol and were included in the data analysis. Recruitment and drop-outs are depicted as a CONSORT [45] flow diagram in Figure 1.

### 3.2. Reporting of Adverse Events

During the course of the trial, no adverse events were reported by the study patients.

### 3.3. Periodontal and General Health Profile

Periodontal and general health-related parameters are shown in Table 1. In the placebo group, the percentage of male study patients was significantly higher than in the test group (67% vs. 33%). All other recorded individual differences were small and statistically not significant.

### 3.4. Percentage of BoP-Positive Sites (Primary Outcome)

The percentage of BoP-positive sites at baseline and at day 90 are depicted in Table 2. At baseline, the mean percentage of BoP-positive sites did not differ significantly between the groups (10.4% vs. 14.2% respectively). At reevaluation, the mean percentage of BoP-positive sites had significantly decreased to 3.0% in the test group (*p* < 0.00014), whereas in the controls, a reduction to 9.4% did not reach the level of significance (*p* < 0.07). The difference between the groups was significant (*p* < 0.017). The calculated effect size for the reduction of BoP was 0.86 ± 0.33 SD.

### 3.5. Secondary Study Outcomes

The data of the secondary outcomes PISA, GI and PCR are shown in Table 3.

### 3.6. Periodontal Inflamed Surface Area (PISA)

At baseline, the recorded mean PISA score did not differ significantly between the groups (test: 170.6 mm^2^, control: 229.4 mm^2^). At day 90, the mean PISA score of the test group was significantly lower compared to baseline (53.7 mm^2^; *p* < 0.00036), whereas the mean PISA score of the control group was not significantly different from baseline (184.3 mm^2^; *p* = 0.3). The difference between the groups at reevaluation (test: 53.7 mm^2^, control: 184.3 mm^2^) was significant (*p* ≤ 0.011).

### 3.7. Gingival Index (GI)

At baseline, the mean GI score was significantly higher in the test group than in the controls (GI 0.5 vs. GI 0.4; *p* ≤ 0.044). The mean GI score at day 90 was significantly reduced compared to baseline in both experimental groups (test: GI 0.1 (*p* ≤ 0.00011)); control: GI 0.3 (*p* ≤ 0.022)). The difference between the groups proved to be significant (*p* ≤ 0.029).

### 3.8. Plaque Control Record (PCR)

Baseline mean PCR scores did not differ significantly between the groups (test: 25.6%; control: 25.4%). At day 90, the mean PCR score was significantly reduced compared to the baseline (test: 9.6% (*p* < 0.00025); control:18.1% (*p* < 0.012)). The difference between the groups did not reach the level of significance (*p* ≤ 0.58).

## 4. Discussion

The results of this controlled clinical trial suggest that the addition of a specific collagen peptide-containing food supplement to the daily diet of periodontal aftercare patients may further improve the anti-inflammatory efficacy of PMPR, as shown by a significantly lower percentage of BoP-positive sites in the test group at reevaluation.

The difference in the primary outcome was also associated with significantly more pronounced improvements in the secondary outcomes of PISA and GI, corresponding to a significantly greater decrease in overall periodontal inflammatory burden.

The reduction in periodontal inflammation observed in the control group after PMPR was well within the range reported in systematic reviews of the efficacy of PMPR in periodontal aftercare [2,5]. Nevertheless, an observed mean percentage of BoP-positive sites of 9.4% at reevaluation is still close to the definition of gingival inflammation according to the current classification [7].

By contrast, the addition of specific collagen peptide consumption to PMPR led to a mean BoP score of only 3.0% at reevaluation. The observed improvement of mean PISA and GI scores in the test group correspond to this situation, with a persisting mean PISA score of only 53.7 mm^2^ compared to 184.3 mm^2^ in the controls. This demonstrates that the anti-inflammatory efficacy of adjuvant collagen peptide consumption in the control of periodontal inflammation is comparable to the benefits of the adjuvant use of antimicrobial agents reported by a recent meta-analysis [46]. 

Mean PCR also decreased significantly between baseline and reevaluation, without significant differences between the groups. This indicates that the observed beneficial impact of collagen peptide consumption on periodontal inflammation is not primarily due to improved plaque control. It rather appears to be based on a systemic immune modulation and/or the resolution of disease-promoting bacterial dysbiosis. This is in line with the findings of other clinical trials evaluating the impact of dietary changes on gingival inflammation [14,15,17,18] and the dysbiosis model of periodontitis development [47].

The proposed modulation of the immune system as well as the presumed resolution of bacterial dysbiosis may be due to the high L-arginine content of the specific collagen peptides used in this study (7.8 g/100 g). The oxidation of L-arginine to L-citrulline by the activity of a nitric oxide synthases (NOS) is a well-known pathway for the formation of nitric oxide (NO), an essential signalling molecule involved in a multitude of physiological processes in the human body. NO formation plays a major role in the development of inflammatory conditions as well as in their resolution and prevention [48]. It reduces, for example, the synthesis of the chemo-attractive protein MCP-1 [49] and inhibits leucocyte adherence to vascular walls as well as platelet aggregation and activation [50]. As far as we know, there have been no previous clinical trials assessing the impact of dietary collagen peptides on periodontal inflammation. However, the results of our study confirm the positive findings of investigations that assessed the anti-inflammatory effect of collagen peptide consumption in patients suffering from chronic osteoarthritis [33,34,35] as well as endothelial dysfunction [51].

In addition to directly modulating the immune response of the host, the presence and formation of NO also has a profound impact on the composition and metabolism of the orodigestive microbiota [18,51,52,53,54,55]. NO is found in significant quantities in dental plaque [56] and has a pronounced inhibitory effect against periodontal pathogens [52,53,57].

An increase in salivary nitrite as an alternative source of physiological NO-formation [58] was accompanied by a resolution of disease-promoting dysbiosis in gingivitis-associated bacterial biofilms [18,59]. In addition, the ingested collagen peptides may also act as a prebiotic. Their microbial fermentation in the colon increases the concentration of tissue-protective antioxidants as well as the concentration of short-chain fatty acids, which are important mediators of lymphocyte maturation [32,60,61].

Relevant limitations of this study are the comparatively short observation period of 3 months without long-term follow-up, the small number of participants and the lack of documentation of dietary behaviour or adherence to a standardised diet. These are in line with the reported restrictions of other clinical trials evaluating the impact of specific dietary intervention [15,21]. They also reflect the fact that it is very difficult to successfully conduct studies involving food restrictions in human volunteers or patients over a long period of time due to a steadily decreasing level of compliance [62].

As mean PCR and GI scores also improved significantly in the placebo group, the impact of a positive Hawthorne effect may not be ruled out. Nevertheless, the significant difference between both experimental groups regarding mean GI scores at day 90 in favor of the test group suggests a significantly more pronounced reduction in inflammation not attributable to improved oral hygiene alone.

Therefore, future studies should aim to recruit larger cohorts of periodontitis patients with a higher inflammatory burden and provide an observation period of at least 6–12 months to test the clinical relevance of the findings obtained in this pilot study. Additionally, the optimal dosage of administration needs to be established. As the effect of a collagen peptide preparation on the microbiota and the host response is decisively influenced by its specific composition [32], the results observed with the consumption of this study preparation may not be transferred to similar preparations with a different composition without further clinical evaluation.

## 5. Conclusions

Supplementing professional plaque control with the daily consumption of specific collagen peptides may have the potential to further improve the outcome of established periodontal aftercare therapy. Due to the limits of the present investigation, however, further research is needed before a general recommendation for daily practice can be given.

## Figures and Tables

**Table 1:** Demographics and general health profile.

Variable	Test Group	Control Group	*p*-Value
	*n* = 20	*n* = 19	
Age (yrs) median (CI)	59.6 (56.6–62.6)	(55.2–63.1)	p_U_ = 0.94
Male gender no. (%)	6 (33%)	12 (67%)	P_c_ = 0.036
No. of teeth median (CI)	25 (22–27)	26 (24–29)	p_U_ = 0.23
PPD baseline (mm) median (CI)	2.9 (2.7–3.1)	2.9 (2.6–3.1)	p_U_ = 0.64
Occasional Smoking (<10 cigarettes/day)	2 (10%)	2 (10.2%)	P_c_ = 1.0
Osteoarthritis	1 (5%)	1 (5.3%)	P_c_ = 1.0
Hypertension	2 (10%)	2 (10.5%)	P_c_ = 1.0
Hypothyroidism	5 (25%)	1 (5.3 %)	P_c_ = 0.18

Median (CI)—median (25%; 75% confidence interval); p_U_ from Mann–Whitney *U* test; P_c_ from chi-square test.

**Table 2:** Percentage of sites being positive for bleeding on probing (BoP).

Visit	Test Group *n* = 20 mean ± SD	Control Group *n* = 19 mean ± SD	p_U_ between Groups
BoP % Baseline	10.4 ± 7.0	14.2 ± 10.3	0.29
BoP % Day 90	3.0 ± 3.8	9.4 ± 9.9	0.017
p_W_ within the groups	0.00014	0.07	

SD: standard deviation; p_U_ (Mann–Whitney *U* test); p_W_ (Wilcoxon signed rank test).

**Table 3:** Periodontal Inflamed Surface Area (PISA), Gingival Index (GI) and Plaque Control Record (PCR).

Visit	Test Group *n* = 20 mean ± SD	Control Group *n* = 19 mean ± SD	p_U_ between Groups
PISA (mm^2^) Baseline	170.6 ± 129.9	229.4 ± 161.6	0.23
PISA (mm^2^) Day 90	53.7 ± 70.5	184.3 ± 214.7	0.011
p_W_ within the groups	0.00036	0.3	
GI Baseline	0.5 ± 0.3	0.4 ± 0.2	0.044
GI Day 90	0.1 ± 0.2	0.3 ± 0.2	0.029
p_w_ within the groups	0.00011	0.022	
PCR% Baseline	25.6 ± 19.5	25.4 ± 14.3	0.81
PCR% Day 90	9.6 ± 10.3	18.1 ± 15.9	0.14
p_w_ within the groups	0.0025	0.012	

SD: standard deviation; p_U_ (Mann–Whitney *U* test); p_W_ (Wilcoxon signed rank test).

## Data Availability

The data supporting the findings of this study are available from the corresponding author upon reasonable request.
